# A Network Meta-Analysis of Two Doses of Recombinant Human Thrombopoietin for Treating Sepsis-Related Thrombocytopenia

**DOI:** 10.1155/2022/2124019

**Published:** 2022-12-30

**Authors:** Dandan Chen, Yu Hou, Chaochao Wei, Xingjun Cai

**Affiliations:** ^1^Department of Critical Care Medicine, Affiliated Haikou Hospital of Xiangya Medical College, Central South University, Haikou, China; ^2^Department of Pulmonary and Critical Care Medicine, Hainan General Hospital, Haikou, China

## Abstract

Previous studies suggest that sepsis remains a common critical illness with a global incidence of 31.5 million. The aim of this study was to evaluate the comparative therapeutic value of recombinant human thrombopoietin (rhTPO) in treating sepsis patients with thrombocytopenia. We conducted a comprehensive electronic search of PubMed, EMBASE, the Cochrane Library, and CNKI from its inception through December 31, 2021. Thirteen randomized controlled trials (RCTs) involving 963 patients were included. Network meta-analyses showed that rhTPO 300 U/kg/day and rhTPO 15000 U/day significantly increased the platelet (PLT) levels on the 7^th^ day and decreased the requirement of transfusion of red blood cells (RBCs), plasma, and PLT compared with IVIG and NAT. SUCRA showed that rhTPO 300 U/kg/day ranked first in terms of 28-day mortality (85.5%) and transfusion, including RBC (88.7%), plasma (89.6%), and PLT (95.2%), while rhTPO 15000 U/day ranked first for the length of the intensive care unit (ICU) stay (95.9%) and PLT level at day 7 (91.6%). rhTPO 300 U/kg/day may be the optimal dose to reduce 28-day mortality and transfusion requirements. However, rhTPO 15000 U/day may be the optimal dose for shortening the ICU stay and increasing the PLT level on the 7th day. However, additional studies to further validate our findings are needed.

## 1. Introduction

Sepsis remains a common critical illness with a global incidence of 31.5 million [1] and the leading cause of death in the intensive care unit (ICU) [2], with an annual mortality rate of 16.8% [3]. Thrombocytopenia [4], defined as sepsis-associated thrombocytopenia, is frequently seen in patients with sepsis, and has been reported to occur in 35%–59% of the patients [5].

Patients with sepsis diagnosed with thrombocytopenia may develop multiple organ dysfunction and have a higher mortality rate [6–8]. Specifically, thrombocytopenia accounts for 13%–83% of the mortality rate in patients with sepsis [9, 10]. Sepsis-associated thrombocytopenia was also found to be strongly associated with longer ICU stays, and the length of hospital stay was a prognostic indicator in patients with sepsis [4]. A previous meta-analysis showed that sepsis-associated thrombocytopenia significantly increases the risk of complications such as shock and acute kidney injury [11]. Therefore, there is an urgent need to develop safe and effective treatment strategies to restore platelet (PLT) levels in septic patients with thrombocytopenia [12].

A series of treatments, such as anti-infective therapy, transfusion of PLT, intravenous injection of recombinant human interleukin (rhIL) including rhIL-6 and rhIL-11, intravenous immunoglobulin (IVIG), and administration of platelet-elevating drugs, are currently available for sepsis-associated thrombocytopenia [13–15]. Because of the scarcity of resources, transfusion-related complications, and PLT antibody production, the clinical application of PLT transfusion is strictly limited to patients with sepsis [16, 17]. The clinical use of intravenous rhIL is associated with mild thrombopoietic activity and unacceptable adverse effects; therefore, the procedure is limited and needs more caution in clinical application [17]. So, the clinical use of IVIG is not recommended for the treatment of sepsis-associated thrombocytopenia [18].

As a full-length glycosylated TPO, recombinant human thrombopoietin (rhTPO) has biological functions similar to endogenous TPO [19]. Studies have shown that rhTPO effectively increases peripheral blood PLT levels in patients with immune- or chemotherapy-related thrombocytopenia and reduces adverse effects [20, 21]. Therefore, rhTPO may be a rescue therapy for septic patients with thrombocytopenia. In addition, a recent meta-analysis demonstrated that in patients with sepsis-associated thrombocytopenia, PLT levels were significantly elevated on the 7^th^ day after administration of rhTPO, and blood product transfusion volumes were reduced [15]. Notably, two different dosing regimens of rhTPO, including rhTPO 300 U/kg/day and rhTPO 15000 U/day, were available for treating septic patients with thrombocytopenia, but which dosing regimen might be better remains unclear [22]. Therefore, the present network meta-analysis aimed to compare the therapeutic values of two dosing regimens of rhTPO.

## 2. Materials and Methods

### 2.1. Study Design

We performed this study according to the preferred reporting items for systematic reviews and meta-analyses (PRISMA) extension statement for reporting network meta-analyses [23]. Ethical approval and informed consent were not required as this was a network meta-analysis of published studies. Moreover, we did not register a formal protocol for this network meta-analysis.

### 2.2. Eligibility Criteria

We designed eligibility criteria based on the PICOS acronym, and studies that met the following criteria were included in this network meta-analysis: (a) participant (P): adult patient diagnosed with sepsis-related thrombocytopenia [24]; (b) intervention (I): rhTPO was prescribed for patients in the study group; (c) comparison (C): patients in the control group were not prescribed additional therapy (NAT) or IVIG in addition to conventional antibiotic therapy (CAT); (d) outcomes (O): reported at least one of the following: 28-day mortality, the length of ICU stay, platelet level on the 7^th^ day, and transfusion of blood products including red blood cells (RBCs), plasma, and platelets; and (e) study design (S): only randomized controlled trials (RCTs) with full texts published in English and Chinese.

Studies that met the following criteria were excluded from this study: (a) ineligible study designs, such as case reports and conference abstracts; (b) replicate studies published by the same author or project; and (c) essential data for synthesis was not available.

### 2.3. Literature Retrieval

A comprehensive search was conducted independently by two researchers (Dandan Chen and Yu Hou) in PubMed, EMBASE, the Cochrane Library, and the China National Knowledge Infrastructure (CNKI) to identify relevant studies published before December 31, 2021. We developed a search query using the following keywords and MeSH terms: “sepsis,” “pyemia,” “pyohemia,” “pyemia,” “septicemia,” “specific infection,” “systemic inflammatory response syndrome,” “SIRS,” “septic shock,” “thrombocytopenia,” “thrombopenia,” “thrombopoietin,” “TPO,” “thrombocytopoiesis stimulating factor,” and “colony-stimulating factors.” We identified additional studies by checking the references of included reviews and eligible studies. Any conflicts between the two researchers were resolved with the help of a third researcher (Xingjun Cai).

### 2.4. Study Selection

After the removal of duplicate records, all titles and abstracts of the remaining studies were independently screened by two researchers (Dandan Chen and Chaochao Wei) for the initial eligibility assessment. Then, the full texts of the remaining studies were retrieved for the final eligibility assessment. With the help of a third researcher, any conflicts were resolved.

### 2.5. Data Extraction

Basic information was independently extracted by two researchers (Dandan Chen and Chaochao Wei) from included studies, including the first author, publication year, sample size, percentage of male participants, age of participants, baseline PLT level, baseline acute physiology, age, chronic health evaluation II/III (APACHE II/III) score, and outcome data. We emailed the leading author to obtain related data when essential data were not available in the original study. Any conflicts were resolved with the help of a third researcher (Xingjun Cai).

### 2.6. Outcomes of Interest

We regarded the 28-day mortality and the length of ICU stay as the primary outcomes, and PLT levels on the 7^th^ day posttreatment and transfusion of blood products including RBC, plasma, and PLT, as the secondary outcome.

### 2.7. Geometry of the Evidence Network

The evidence structure for each outcome was displayed using a network plot. In the network plot, the size of the node is weighted by the accumulated sample size, marked as white numerical values, and the width of the solid line is weighted by the number of direct comparisons, marked as a black numerical value close to the solid line [25]. Furthermore, the dotted line indicates the lack of direct comparison between the two interventions.

### 2.8. Risk of Bias Assessment

Two independent researchers (Dandan Chen and Yu Hou) assessed the risks of bias of each study from the following seven items, according to the Cochrane risk of bias assessment tool [26]: random sequence generation, allocation concealment, blinding of participants and personnel, blinding of outcomes assessment, incomplete outcome data, selective reporting, and other bias. Based on the evaluation criteria, each item was rated as “low,” “unclear,” or “high” risk. Any conflicts were resolved with the help of a third researcher.

### 2.9. Statistical Analysis

The odds ratio (OR) with a 95% confidence interval (CI) was used to express the pooled result for 28-day mortality, and the mean difference (MD) with 95%CI was used to express the differences in the length of ICU stay, PLT levels on the 7^th^ day posttreatment, and transfusion of RBC, plasma, and PLT. The transitivity of included studies was assessed based on clinical and methodological characteristics [27, 28]. Consistency between direct and indirect effects was assessed based on the global consistency model test [29] and the local consistency model test [30]. Meanwhile, the node-splitting method was used to check whether there was an inconsistency in the closed loop [31, 32]. A random effect model was used to calculate the relative efficacy of different doses [33], and a forest plot was used to show the differences between interventions [34]. The surface under the cumulative ranking (SUCRA) plot was used to show the ranking of different interventions in the same outcome [35]. Publication bias was checked based on the comparison-adjusted funnel plot [36]. All analyses were performed using STATA 14.0 (StataCorp LP, College Station, Texas, USA) with the “network” command [37]. *p* < 0.05 was considered to be a statistical difference.

## 3. Results

### 3.1. Literature Selection

**Figure 1:**
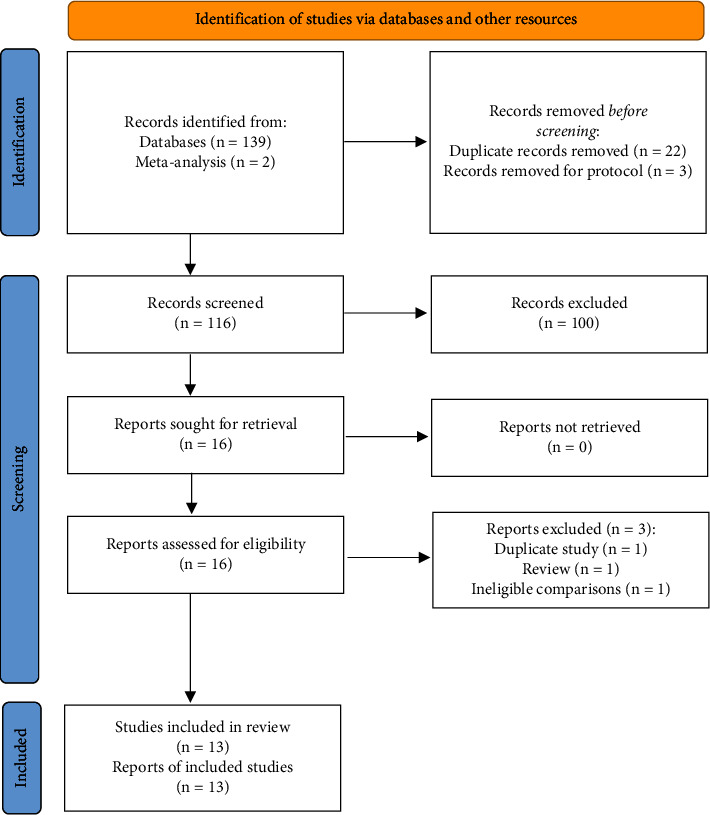
PRISMA flow diagram of study selection.

A total of 139 relevant studies were identified from the initial literature retrieval, and after removing duplicate records (*n* = 25) and irrelevant studies (*n* = 100), 14 articles were retained for further eligibility assessment. After the screening of full texts, 11 studies were identified as meeting the eligibility criteria. In addition, 2 eligible studies were added from the published meta-analysis. Finally, this network meta-analysis included 13 studies [38–50] involving 963 patients. The details of the study selection are shown in Figure 1.

### 3.2. Study Characteristics

All 13 studies were reported by Chinese researchers between 2011 and 2021. The sample size ranged from 43 to 102, with a cumulative number of 963. Two studies [40, 44] compared rhTPO 15000 U/day with IVIG; 4 studies [38, 41, 42, 46] compared rhTPO 300 U/kg/day with IVIG; 3 studies [39, 45, 50] compared rhTPO 15000 U/day with NAT; and 4 studies [43, 47–49] compared rhTPO 300 U/kg/day with NAT. Additional characteristics of the included studies are shown in Table 1. The results of individual studies are shown in Table S1.

### 3.3. Risk of Bias

Figure S1 shows details of the risk of bias for the 13 eligible studies. All 13 studies [38–50] used appropriate methods to generate random sequences, but only 2 studies [46, 49] explicitly reported the methods to perform allocation concealment. Risks were unclear in all studies with respect to the blinding of participants, personnel, and outcome assessment [38–50]. All studies [38–50] had a low risk of incomplete outcome data and selective reporting of outcomes. Furthermore, for other biases, the risk of all studies was unclear [38–50].

### 3.4. Transitivity Assessment

We conducted a transitivity assessment between comparisons based on five main characteristics, including sample size, the proportion of males, mean age, baseline PLT levels, and APACHE scores. As shown in Table S2, transitivity was determined for most of the comparisons, except for rhTPO 15000 U/kg/day vs. NAT (*p* = 0.034) and rhTPO 300 U/day vs. NAT (*p* = 0.042) for male proportion and IVIG vs. NAT (*p* = 0.003) for disease severity.

### 3.5. 28-Day Mortality

**Figure 2:**
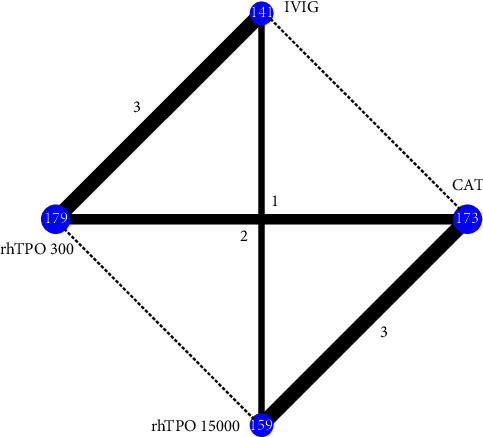
Evidence network of 28-day mortality. NAT, no additional treatment; IVIG, intravenous immunoglobulin; rhTPO 300, 300 U/kg/d recombinant human thrombopoietin; rhTPO 15000, 15000 U/d recombinant human thrombopoietin.

**Figure 3:**
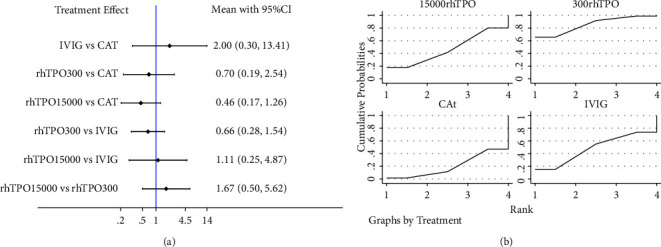
Network meta-analysis of the relative efficacy (a) and the rank probabilities (b) among different treatment strategies in terms of 28-day mortality. NAT, no additional treatment; IVIG, intravenous immunoglobulin; rhTPO 300, 300 U/kg/d recombinant human thrombopoietin; rhTPO 15000, 15000 U/d recombinant human thrombopoietin.

For 28-day mortality, a network plot of the evidence structure is shown in Figure 2. Global and local consistency model tests showed no inconsistency (Figure S2), and the consistency model was used for network meta-analysis. No significant difference was found between treatment strategies (Figure 3(a)). The results of SUCRA showed that rhTPO 300 U/day had the highest probability of being the best (85.5%), followed by rhTPO 15000 U/kg/day (46.4%) (Figure 3(b)).

### 3.6. The Length of ICU Stay

**Figure 4:**
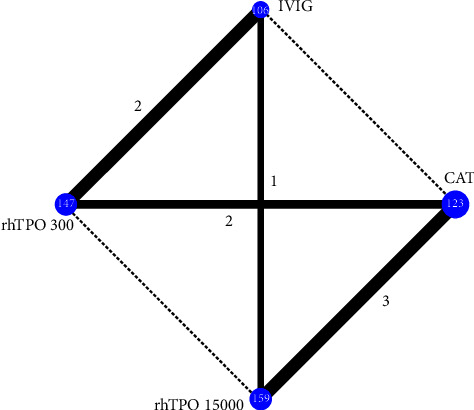
Evidence network of the length of ICU stay. NAT, no additional treatment; IVIG, intravenous immunoglobulin; rhTPO 300, 300 U/kg/d recombinant human thrombopoietin; rhTPO 15000, 15000 U/d recombinant human thrombopoietin.

**Figure 5:**
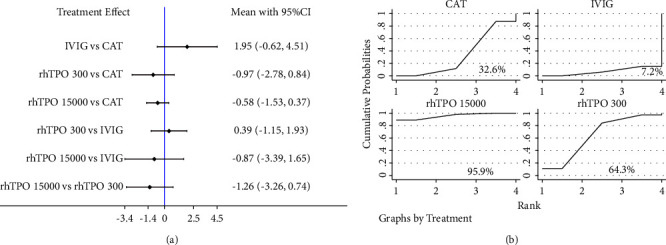
Network meta-analysis of the relative efficacy (a) and the rank probabilities (b) among different treatment strategies in terms of the length of ICU stay. NAT, no additional treatment; IVIG, intravenous immunoglobulin; rhTPO 300, 300 U/kg/d recombinant human thrombopoietin; rhTPO 15000, 15000 U/d recombinant human thrombopoietin.

For the length of the ICU stay, a network plot of the evidence structure is shown in Figure 4. Global and local consistency model tests showed no inconsistency (Figure S3), so the consistency model was chosen. No significant difference was detected between treatment strategies (Figure 5(a)). The results of SUCRA showed that rhTPO 1500 U/kg/day had the highest probability of being the best (95.9%), followed by rhTPO 300 U/day (64.3%) (Figure 3(b)).

### 3.7. PLT Level on the 7^th^ Day

**Figure 6:**
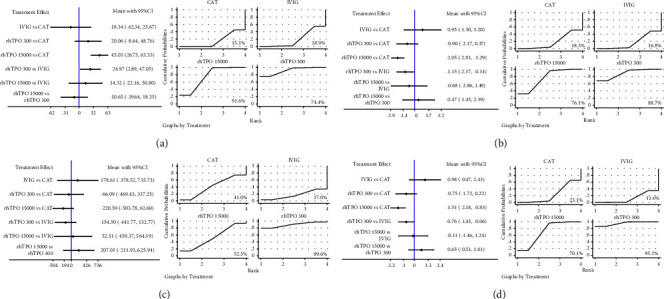
Network meta-analysis of the relative efficacy and the rank probabilities among different treatment strategies in terms of the level of platelet on the 7^th^ day (a), transfusion of RBC (b), transfusion of plasma (c), and transfusion of platelets (d). NAT, no additional treatment; IVIG, intravenous immunoglobulin; rhTPO 300, 300 U/kg/d recombinant human thrombopoietin; rhTPO 15000, 15000 U/d recombinant human thrombopoietin.

For the PLT level on the 7^th^ day posttreatment, a network plot of the evidence structure is shown in Figure S4a. Global and local consistency model tests showed no inconsistency (Figure S5a), so the consistency model was chosen. The pooled results showed that rhTPO 300 U/day and rhTPO 15000 U/kg/day significantly increased PLT levels on the 7^th^ day posttreatment compared with IVIG and NAT (Figure 6(a)). The results of SUCRA showed that rhTPO 1500 U/kg/day had the highest probability of being the best (91.6%), followed by rhTPO 300 U/day (74.4%).

### 3.8. Transfusion of Blood Products

For the transfusion of RBCs, a network plot of the evidence structure is shown in Figure S4a. Global and local consistency model tests showed no inconsistency (Figure S5b), so the consistency model was selected. Pooled results showed that rhTPO 300 U/day and rhTPO 15000 U/kg/day were associated with lower RBC transfusions compared with IVIG and NAT, respectively (Figure 6(b)). The results of SUCRA showed that rhTPO 300 U/day had the highest probability of being the best (88.7%), followed by rhTPO 15000 U/kg/day (76.1%).

For the transfusion of plasma, a network plot of the evidence structure is shown in Figure S4c. Global and local consistency model tests showed no inconsistency (Figure S5c), and a consistency model was selected. No significant difference was detected between treatment strategies (Figure 6(c)). The results of SUCRA showed that rhTPO 300 U/day had the highest probability of being the best (89.6%), followed by rhTPO 15000 U/kg/day (52.5%).

For the transfusion of PLT, a network plot of the evidence structure is shown in Figure S4d. Global and local consistency model tests indicated no inconsistency (Figure S5d), and the consistency model was selected. The pooled results showed that rhTPO 300 U/day and rhTPO 15000 U/kg/day were associated with lower PLT transfusions compared with IVIG and NAT, respectively (Figure 6(d)). The results of SUCRA indicated that rhTPO 300 U/day had the highest probability of being the best (95.2%), followed by rhTPO 15000 U/kg/day (70.1%).

### 3.9. Closed-Loop Inconsistency and Publication Bias

For each outcome, the closed-loop inconsistency was also evaluated based on the node-splitting method. As shown in Figure S6, no closed-loop inconsistency was found, indicating the robustness of all pooled results. In addition, the publication bias of the primary outcomes was further examined. As shown in Figure S7, the symmetric outline of the comparison-adjusted funnel plots indicated that there was no publication bias.

## 4. Discussion

Evidence suggests that patients with sepsis-related thrombocytopenia have longer ICU stays [4] and a worse prognosis [8, 10]. As a novel rescue therapy, rhTPO has been shown to be effective in increasing peripheral PLT levels [19], as confirmed by a recent pairwise meta-analysis [15]. Unfortunately, which doses of rhTPO might be optimal for septic patients with thrombocytopenia remains unclear as a direct comparison is absent. Therefore, in order to draw firm conclusions, this study indirectly investigated the comparative therapeutic values of two available doses of rhTPO by introducing a network meta-analysis. Based on the results of this network meta-analysis, rhTPO 300 U/day may be the best option for reducing 28-day mortality and blood transfusion requirements. However, rhTPO 15000 U/kg/day may be the best option for shortening the ICU stay and increasing peripheral PLT levels on the 7th day posttreatment.

Notably, a pairwise meta-analysis [15] determined whether rhTPO is a beneficial strategy in septic patients with thrombocytopenia. Based on pooled results from 10 eligible RCTs, rhTPO was associated with increased PLT levels on the 7^th^ day posttreatment and decreased blood product transfusions during hospitalization. Unfortunately, the optimal dose of rhTPO was not determined in this meta-analysis, which greatly confounds clinical decision-making. Furthermore, this meta-analysis missed an eligible study [38] that investigated the therapeutic values between rhTPO 300 U/day and IVIG. Furthermore, since the publication of this meta-analysis, 2 additional eligible studies have been provided. In contrast to the previous meta-analysis, the present study included all available studies to determine the therapeutic values of 2 different doses of rhTPO by introducing a network meta-analysis technique. Therefore, the optimal dose for each outcome was determined based on more robust and reliable results.

This network meta-analysis yielded some robust findings due to 3 methodological strengths: (a) creative use of the network meta-analysis to determine the comparative therapeutic values between two different doses of rhTPO that were not directly compared in the original study; (b) SUCRA plots based on ranking probabilities were used to determine the optimal dose for each clinical outcome; and (c) this network meta-analysis included both control strategies including no additional treatment and IVIG, to increase statistical power.

Certainly, some limitations may have negatively impacted our findings: (a) the sample size is insufficient, because although 13 RCTs were included in this network meta-analysis, only 963 participants were accumulated; (b) all 13 RCTs did not explicitly describe whether participants, personnel, and outcomes assessment were blinded, which could lead to biased implementation; (c) all 13 studies did not report protocol registration and conflict of interest, which could be a source of bias; (d) all 13 studies were conducted in China, so the results should be cautiously used with caution in different clinical settings; (e) although we conducted this network meta-analysis strictly in accordance with the methodological framework recommended by the Cochrane handbook, a formal public protocol was not available for the current network meta-analysis, which will inevitably negatively affect the transparency of this network meta-analysis; (f) due to limited data, we did not assess other outcomes such as the length of activated partial thromboplastin time (APTT) and prothrombin time (PT) on day 7, which may be negative for the comprehensiveness of our findings; (g) one study used APACHE III for severity assessment, and it differed from other eligible studies which used APACHE II for severity assessment, which might be the source of bias; and (h) we confirmed transitivity assumption among most of the available comparisons; however, 3 of these comparisons differed significantly in male proportion and disease severity, which may inevitably compromise the reliability of our findings because subgroup analysis cannot be performed due to limited studies.

## 5. Conclusion

This network meta-analysis suggests that rhTPO 300 U/day may be the best option for improving 28-day mortality and transfusion of blood products for the treatment of septic patients with thrombocytopenia. However, rhTPO 15000 U/kg/day may be the best option for shortening the ICU stay and increasing PLT levels on the 7^th^ day posttreatment. However, given the limitations, we recommend more studies to further validate our findings.

## Figures and Tables

**Table 1:** Baseline characteristics of included studies.

Study	Sample size	Proportion of male patients, %	Age, years	The level of PLT, 10^9^/L	APACHE II	Interventions	
						rhTPO	Comparison
Feng, et al., 2018 [40]	52 vs 25	59.6 vs 64.0	(51.19 ± 18.17) vs (49.62 ± 20.54)	(42.19 ± 8.6) vs (43.07 ± 10.01)	(23.5 ± 3.7) vs (22.6 ± 2.9)	15000 U/d	400 mg/kg IVIG
Chen, 2015 [38]	45 vs 45	57.8 vs 64.4	(53.9 ± 11.6) vs (53.5 ± 11.7)	<50	n.a.	300 U/kg/d	400 mg/kg IVIG
Li, 2015 [42]	32 vs 35	62.5 vs 45.7	(58.56 ± 25.43) vs (59.09 ± 23.89)	(36.93 ± 5.50) vs (35.26 ± 4.71)	(26.94 ± 5.74) vs (24.03 ± 6.35)	300 U/kg/d	400 mg/kg IVIG
Peng, et al., 2021 [44]	45 vs 45	53.3 vs 44.4	(43.35 ± 4.45) vs (46.18 ± 4.72)	(7.47 ± 1.22) vs (7.58 ± 1.04)	(63.45 ± 6.75) vs (65.48 ± 6.12)	15000 U/d	400 mg/kg IVIG
Qi, et al., 2016 [45]	30 vs 30	53.3 vs 50.0	(50.3 ± 26.2) vs (50.9 ± 25.7)	(52.83 ± 16.32) vs (52.11 ± 16.29)	(18.8 ± 2.7) vs (18.1 ± 2.2)	15000 U/d	NAT
Li, et al., 2013 [43]	28 vs 20	53.9	53.49 ± 17.41	n.a.	n.a.	300 U/kg/d	NAT
Dong, et al., 2020 [39]	50 vs 50	60.0 vs 52.0	(56.48 ± 13.58) vs (57.92 ± 12.69)	(39.08 ± 22.15) vs (38.51 ± 20.96)	(14.82 ± 7.74) vs (17.04 ± 7.38)	15000 U/d	NAT
Zhang, et al., 2018 [50]	34 vs 42	61.8 vs 54.8	(54.50 ± 19.53) vs (53.65 ± 15.52)	(30.64 ± 10.19) vs (37.17 ± 1.68)	(20.21 ± 7.10) vs (19.78 ± 6.05)	15000 U/d	NAT
Zhang, et al., 2016 [49]	35 vs 31	48.6 vs 48.4	(56 ± 9) vs (54 ± 8)	(37 ± 8) vs (38 ± 19)	(17 ± 3) vs (17 ± 3)	300 U/kg/d	NAT
Gao, et al., 2011 [41]	21 vs 22	66.7 vs 63.6	(43.10 ± 21.25) vs (41.74 ± 17.65)	(25.14 ± 7.09) vs (26.13 ± 7.11)	(21.93 ± 8.34) vs (23.47 ± 10.26)	300 U/kg/d	400 mg/kg IVIG
Yang, et al., 2015 [48]	30 vs 30	53.3	45.2 ± 12.7	(34.98 ± 0.64) vs (34.31 ± 0.78)	n.a.	300 U/kg/d	NAT
Yan, et al., 2019 [47]	42 vs 42	54.8 vs 52.4	(59.13 ± 0.37) vs (59.14 ± 0.39)	(25.49 ± 2.53) vs (25.52 ± 2.51)	(18.35 ± 2.14) vs (18.31 ± 2.16)	300 U/kg/d	NAT
Wang, et al., 2019 [46]	63 vs 39	61.9 vs 61.5	(57.2 ± 21.2) vs (56.9 ± 18.3)	(28.7 ± 9.7) vs (27.5 ± 14.1)	(22.6 ± 6.1) vs (23.0 ± 4.6)	300 U/kg/d	400 mg/kg IVIG

APACHE II, acute physiology, age, chronic health evaluation II; PLT, platelet; rhTPO, recombinant human thrombopoietin; IVIG; intravenous immunoglobulin; NAT, no additional treatment; n.a., not applicable. ^*∗*^APACHE III for severity assessment.

## Data Availability

All data generated or analyzed during this study are included in this published article/as supplementary information files.

## Supplementary Materials

Supplementary Materials Search strategy of pubmed, search strategy of Embase, and search strategy of Cochrane library are given. *Table S1.* Results of individual studies included in this network meta-analysis. T*able S2.* Transitivity between different comparisons based on major characteristics. *Figure S1.* Risk of bias summary (a) and graph (b). Red (−), yellow (?), and green (+) color indicates high, unclear, and low risk of bias, respectively. *Figure S2*. Consistency model test of 28-day mortality. NAT, no additional treatment; IVIG, intravenous immunoglobulin; rhTPO 300, 300 U/kg/d recombinant human thrombopoietin; rhTPO 15000, 15000 U/d recombinant human thrombopoietin. *Figure S3.* Consistency model test of the length of ICU stay. NAT, no additional treatment; IVIG, intravenous immunoglobulin; rhTPO 300, 300 U/kg/d recombinant human thrombopoietin; rhTPO 15000, 15000 U/d recombinant human thrombopoietin. *Figure S4.* Evidence network of secondary outcomes including the level of platelet on the 7^th^ day (a), transfusion of RBC (b), transfusion of plasma (c), and transfusion of platelet (d). NAT, no additional treatment; IVIG, intravenous immunoglobulin; rhTPO 300, 300 U/kg/d recombinant human thrombopoietin; rhTPO 15000, 15000 U/d recombinant human thrombopoietin. *Figure S5.* The consistency model test of the secondary outcomes including the level of platelet on the 7^th^ day (a), transfusion of RBC (b), transfusion of plasma (c), and transfusion of platelet (d). NAT, no additional treatment; IVIG, intravenous immunoglobulin; rhTPO 300, 300 U/kg/d recombinant human thrombopoietin; rhTPO 15000, 15000 U/d recombinant human thrombopoietin. *Figure S6*. Results of consistency test based on closed-loop. NAT, no additional treatment; IVIG, intravenous immunoglobulin; rhTPO 300, 300 U/kg/d recombinant human thrombopoietin; rhTPO 15000, 15000 U/d recombinant human thrombopoietin; IF, inconsistency factor; CI, confidence interval. *Figure S7.* Comparison-adjusted funnel plot of 28-day mortality (a) and the length of ICU stay (b). (a–d) NAT, IVIG, rhTPO 300, and rhTPO 15000, respectively. NAT, no additional treatment; IVIG, intravenous immunoglobulin; rhTPO 300, 300 U/kg/d recombinant human thrombopoietin; rhTPO 15000, 15000 U/d recombinant human thrombopoietin.
