# Supplementary material for: A Network Meta-Analysis of Two Doses of Recombinant Human Thrombopoietin for Treating Sepsis-Related Thrombocytopenia
Source: Int J Clin Pract. 2022 Dec 30;2022:2124019. doi: 10.1155/2022/2124019 (PMC9822753; doi:10.1155/2022/2124019)
Supplement: Supplementary Materials — Search strategy of pubmed, search strategy of Embase, and search strategy of Cochrane library are given. Table S1. Results of individual studies included in this network meta-analysis. Table S2. Transitivity between different comparisons based on major characteristics. Figure S1. Risk of bias summary (a) and graph (b). Red (−), yellow (?), and green (+) color indicates high, unclear, and low risk of bias, respectively. Figure S2. Consistency model test of 28-day mortality. NAT, no additional treatment; IVIG, intravenous immunoglobulin; rhTPO 300, 300 U/kg/d recombinant human thrombopoietin; rhTPO 15000, 15000 U/d recombinant human thrombopoietin. Figure S3. Consistency model test of the length of ICU stay. NAT, no additional treatment; IVIG, intravenous immunoglobulin; rhTPO 300, 300 U/kg/d recombinant human thrombopoietin; rhTPO 15000, 15000 U/d recombinant human thrombopoietin. Figure S4. Evidence network of secondary outcomes including the level of platelet on the 7th day (a), transfusion of RBC (b), transfusion of plasma (c), and transfusion of platelet (d). NAT, no additional treatment; IVIG, intravenous immunoglobulin; rhTPO 300, 300 U/kg/d recombinant human thrombopoietin; rhTPO 15000, 15000 U/d recombinant human thrombopoietin. Figure S5. The consistency model test of the secondary outcomes including the level of platelet on the 7th day (a), transfusion of RBC (b), transfusion of plasma (c), and transfusion of platelet (d). NAT, no additional treatment; IVIG, intravenous immunoglobulin; rhTPO 300, 300 U/kg/d recombinant human thrombopoietin; rhTPO 15000, 15000 U/d recombinant human thrombopoietin. Figure S6. Results of consistency test based on closed-loop. NAT, no additional treatment; IVIG, intravenous immunoglobulin; rhTPO 300, 300 U/kg/d recombinant human thrombopoietin; rhTPO 15000, 15000 U/d recombinant human thrombopoietin; IF, inconsistency factor; CI, confidence interval. Figure S7. Comparison-adjusted funnel plot of 28-day mortality (a) and the len [file 2124019.f1.zip › Figure S5.pdf]

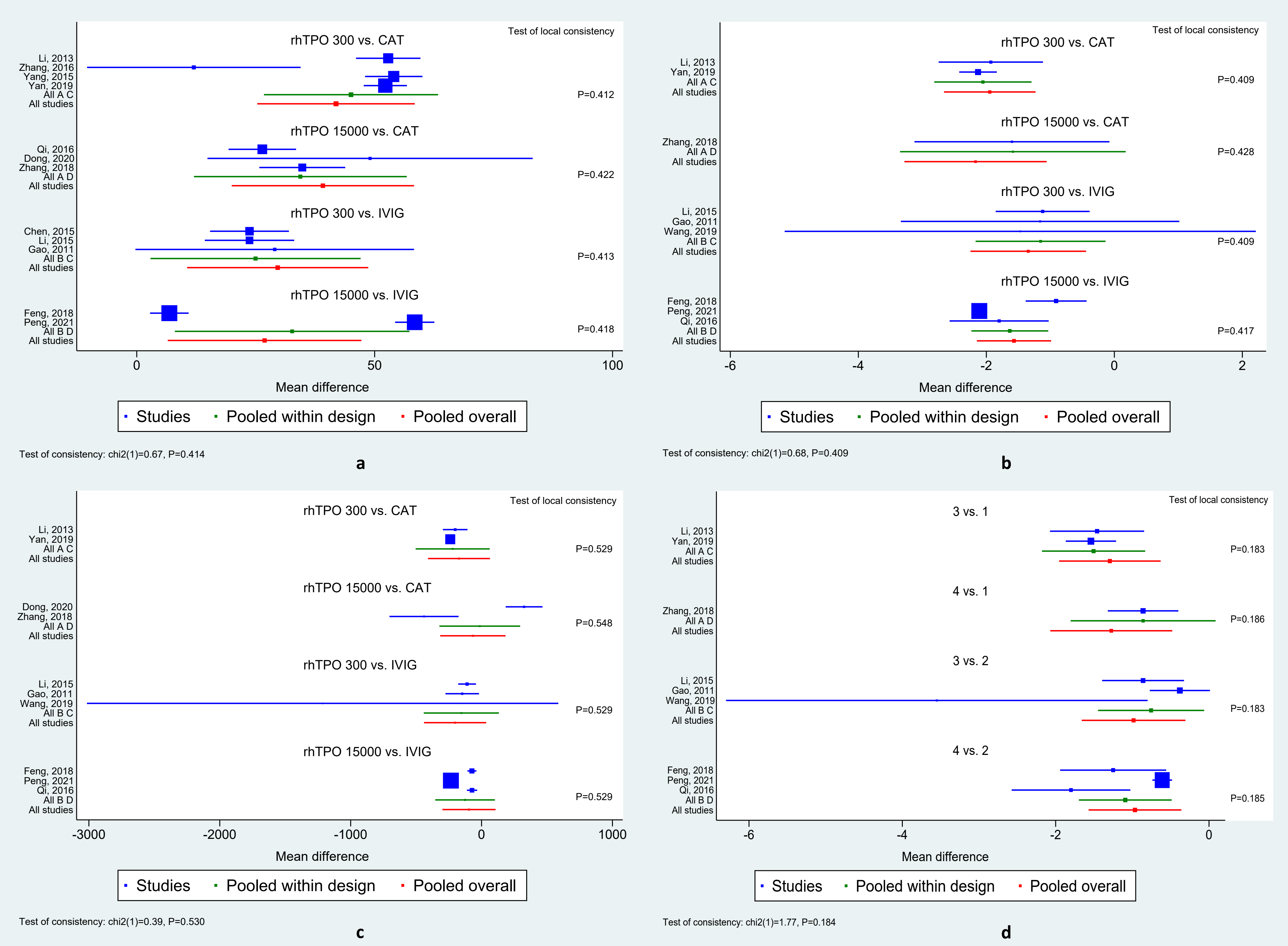

Test of local consistency
rhTPO 300 vs. CAT
Li, 2013
Zhang, 2016
Yang, 2015
Yan, 2019
All A C
All studies
P=0.412
rhTPO 15000 vs. CAT
Qi, 2016
Dong, 2020
Zhang, 2018
All A D
All studies
P=0.422
rhTPO 300 vs. IVIG
Chen, 2015
Li, 2015
Gao, 2011
All B C
All studies
P=0.413
rhTPO 15000 vs. IVIG
Feng, 2018
Peng, 2021
All B D
All studies
P=0.418
0
50
100
Mean difference
Studies
Pooled within design
Pooled overall
Test of consistency: chi2(1)=0.67, P=0.414
a
Test of local consistency
rhTPO 300 vs. CAT
Li, 2013
Yan, 2019
All A C
All studies
P=0.409
rhTPO 15000 vs. CAT
Zhang, 2018
All A D
All studies
P=0.428
rhTPO 300 vs. IVIG
Li, 2015
Gao, 2011
Wang, 2019
All B C
All studies
P=0.409
rhTPO 15000 vs. IVIG
Feng, 2018
Peng, 2021
Qi, 2016
All B D
All studies
P=0.417
-6
-4
-2
0
2
Mean difference
Studies
Pooled within design
Pooled overall
Test of consistency: chi2(1)=0.68, P=0.409
b
Test of local consistency
rhTPO 300 vs. CAT
Li, 2013
Yan, 2019
All A C
All studies
P=0.529
rhTPO 15000 vs. CAT
Dong, 2020
Zhang, 2018
All A D
All studies
P=0.548
rhTPO 300 vs. IVIG
Li, 2015
Gao, 2011
Wang, 2019
All B C
All studies
P=0.529
rhTPO 15000 vs. IVIG
Feng, 2018
Peng, 2021
Qi, 2016
All B D
All studies
P=0.529
-3000
-2000
-1000
0
1000
Mean difference
Studies
Pooled within design
Pooled overall
Test of consistency: chi2(1)=0.39, P=0.530
c
Test of local consistency
3 vs. 1
Li, 2013
Yan, 2019
All A C
All studies
P=0.183
4 vs. 1
Zhang, 2018
All A D
All studies
P=0.186
3 vs. 2
Li, 2015
Gao, 2011
Wang, 2019
All B C
All studies
P=0.183
4 vs. 2
Feng, 2018
Peng, 2021
Qi, 2016
All B D
All studies
P=0.185
-6
-4
-2
0
Mean difference
Studies
Pooled within design
Pooled overall
Test of consistency: chi2(1)=1.77, P=0.184
d
