# Supplementary material for: A Network Meta-Analysis of Two Doses of Recombinant Human Thrombopoietin for Treating Sepsis-Related Thrombocytopenia
Source: Int J Clin Pract. 2022 Dec 30;2022:2124019. doi: 10.1155/2022/2124019 (PMC9822753; doi:10.1155/2022/2124019)
Supplement: Supplementary Materials — Search strategy of pubmed, search strategy of Embase, and search strategy of Cochrane library are given. Table S1. Results of individual studies included in this network meta-analysis. Table S2. Transitivity between different comparisons based on major characteristics. Figure S1. Risk of bias summary (a) and graph (b). Red (−), yellow (?), and green (+) color indicates high, unclear, and low risk of bias, respectively. Figure S2. Consistency model test of 28-day mortality. NAT, no additional treatment; IVIG, intravenous immunoglobulin; rhTPO 300, 300 U/kg/d recombinant human thrombopoietin; rhTPO 15000, 15000 U/d recombinant human thrombopoietin. Figure S3. Consistency model test of the length of ICU stay. NAT, no additional treatment; IVIG, intravenous immunoglobulin; rhTPO 300, 300 U/kg/d recombinant human thrombopoietin; rhTPO 15000, 15000 U/d recombinant human thrombopoietin. Figure S4. Evidence network of secondary outcomes including the level of platelet on the 7th day (a), transfusion of RBC (b), transfusion of plasma (c), and transfusion of platelet (d). NAT, no additional treatment; IVIG, intravenous immunoglobulin; rhTPO 300, 300 U/kg/d recombinant human thrombopoietin; rhTPO 15000, 15000 U/d recombinant human thrombopoietin. Figure S5. The consistency model test of the secondary outcomes including the level of platelet on the 7th day (a), transfusion of RBC (b), transfusion of plasma (c), and transfusion of platelet (d). NAT, no additional treatment; IVIG, intravenous immunoglobulin; rhTPO 300, 300 U/kg/d recombinant human thrombopoietin; rhTPO 15000, 15000 U/d recombinant human thrombopoietin. Figure S6. Results of consistency test based on closed-loop. NAT, no additional treatment; IVIG, intravenous immunoglobulin; rhTPO 300, 300 U/kg/d recombinant human thrombopoietin; rhTPO 15000, 15000 U/d recombinant human thrombopoietin; IF, inconsistency factor; CI, confidence interval. Figure S7. Comparison-adjusted funnel plot of 28-day mortality (a) and the len [file 2124019.f1.zip › Supplementary Tables.docx]

Table S1. Results of individual studies included in this network meta-analysis.

| Study | Comparison | 28-day mortality | Length of ICU stay, day | The level of PLT on the 7^th^ day, ×10^9^/L | Transfusion of RBC, U | Transfusion of plasma, mL | Transfusion of platelet, U |
| --- | --- | --- | --- | --- | --- | --- | --- |
| Feng, et al., 2018 | rhTPO 15000 vs IVIG | n.a. | n.a. | (92.73±9.98) vs (85.88±7.69) | (2.75±1.07) vs (3.66±0.96) | (235.97±80.52) vs (309.53±66.78) | (2.07±1.84) vs (3.32±1.22) |
| Chen, 2015 | rhTPO 300 vs IVIG | n.a. | n.a. | (124.36±26.51) vs (100.66±10.09) | n.a. | n.a. | n.a. |
| Li, 2015 | rhTPO 300 vs  IVIG | 1/32 vs 5/35 | n.a. | (124.35±26.50) vs (100.65±10.08) | (1.35±1.50) vs (2.47±1.63) | (116.13±124.09) vs (226.67±163.87) | (0.84±1.13) vs (1.70±1.15) |
| Peng, et al., 2021 | rhTPO 15000 vs IVIG | 9/45 vs 14/45 | (10.64±1.04) vs (13.46±1.32) | (135.68±12.19) vs (77.26±7.13) | (1.01±0.14) vs (3.12±0.29) | (112.15±9.68) vs (346.15±28.12) | (1.62±0.25) vs (2.23±0.36) |
| Qi, et al., 2016 | rhTPO 15000 vs NAT | 6/30 vs 12/30 | (81.5±3.51) vs (10.12±5.13) | (107.55±6.23) vs (81.16±18.82) | (1.5±1.2) vs (3.3±1.8) | (60.5±40.3) vs (133.3±101.8) | (1.5±1.2) vs (3.3±1.8) |
| Li, et al., 2013 | rhTPO 300 vs NAT | 5/28 vs 11/20 | (11.57±2.33) vs (12.40±2.68) | (103.21±16.07) vs (50.36±7.38) | (1.07±1.27) vs (3.00±1.52) | (128.57±165.23) vs (330.00±162.55) | (0.79±1.07) vs (2.25±1.07) |
| Dong, et al., 2020 | rhTPO 15000 vs NAT | 34/50 vs 33/50 | (12.66±8.94) vs (12.76±12.24) | (125.85±112.3) vs (76.81±50.87) | n.a. | (525±356.3) vs (200±362.5) | n.a. |
| Zhang, et al., 2018 | rhTPO 15000  vs NAT | 12/34 vs 16/42 | (13.21±5.90) vs (16.20±11.30) | (107.36±17.35) vs (72.57±22.78) | (1.5±2.5) vs (3.1±4.2) | (371.43±447.95) vs (810.87±716.52) | (1.00±0.78) vs (1.86±1.25) |
| Zhang, et al., 2016 | rhTPO 300 vs NAT | 8/35 vs 7/31 | (9.8±2.3) vs (10.2±2.8) | (102±48) vs (90±45) | n.a. | n.a. | n.a. |
| Gao, et al., 2011 | rhTPO 300 vs IVIG | 7/21 vs 9/22 | (10.25±2.59) vs (9.41±3.12) | (119.94±58.60) vs (90.94±36.23) | (3.29±3.73) vs (4.45±3.54) | (152.38±199.05) vs (300.00±228.86) | (0.48±0.60) vs (0.86±0.71) |
| Yang, et al., 2015 | rhTPO 300 vs NAT | n.a. | n.a. | (105.21±15.12) vs (51.20±7.45) | n.a. | n.a. | (1.09±0.72) vs (3.25±1.20) |
| Yan, et al., 2019 | rhTPO 300 vs NAT | n.a. | n.a. | (122.52±11.34) vs (70.28±9.85) | (1.04±0.55) vs (3.17±0.80) | (115.36±25.17) vs （354.52±41.48） | (0.73±0.37) vs (2.27±1.02) |
| Wang, et al., 2019 | rhTPO 300 vs IVIG | 20/63 vs 14/39 | (14.9±10.6) vs (16.4±7.4) | n.a. | (10.17±10.39) vs  (11.64±8.41) | (3515.67±3884.05) vs (4730.0±4855.7) | (5.02±6.10) vs (8.57±7.33) |

PLT, platelet; RBC, red blood cell; rhTPO, recombinant human thrombopoietin; IVIG; intravenous immunoglobulin; NAT, no additional treatment; n.a., not applicable.

Table S2. Transitivity between different comparisons based on major characteristics.

| Major variables | Comparisons | MD | SE | p-value |
| --- | --- | --- | --- | --- |
| Sample size, n | rhTPO 15000 vs IVIG | 6.63 | 6.06 | 0.302 |
|  | rhTPO 15000 vs NAT | 6.23 | 5.89 | 0.315 |
|  | rhTPO 300 vs IVIG | 1.33 | 6.42 | 0.839 |
|  | rhTPO 300 vs NAT | 0.93 | 6.10 | 0.881 |
|  | IVIG vs NAT | -0.40 | 5.51 | 0.943 |
| Proportion of males, % | rhTPO 15000 vs IVIG | 10.00 | 10.08 | 0.347 |
|  | rhTPO 15000 vs NAT | 5.49 | 1.98 | 0.034 |
|  | rhTPO 300 vs IVIG | 9.84 | 8.06 | 0.246 |
|  | rhTPO 300 vs NAT | 5.32 | 2.26 | 0.042 |
|  | IVIG vs NAT | -4.51 | 9.04 | 0.638 |
| Mean ages, years | rhTPO 15000 vs IVIG | 4.49 | 7.65 | 0.571 |
|  | rhTPO 15000 vs NAT | -2.31 | 2.80 | 0.429 |
|  | rhTPO 300 vs IVIG | 6.65 | 6.21 | 0.305 |
|  | rhTPO 300 vs NAT | -0.15 | 2.80 | 0.958 |
|  | IVIG vs NAT | -6.80 | 6.41 | 0.312 |
| Baseline PLT levels, 10^9^/L | rhTPO 15000 vs IVIG | -0.08 | 9.49 | 0.993 |
|  | rhTPO 15000 vs NAT | -0.26 | 7.99 | 0.975 |
|  | rhTPO 300 vs IVIG | -0.49 | 6.76 | 0.944 |
|  | rhTPO 300 vs NAT | -0.67 | 4.42 | 0.883 |
|  | IVIG vs NAT | -0.18 | 7.37 | 0.981 |
| Severity assessment^*^ | rhTPO 15000 vs IVIG | -0.12 | 0.06 | 0.088 |
|  | rhTPO 15000 vs NAT | 0.04 | 0.06 | 0.468 |
|  | rhTPO 300 vs IVIG | -0.04 | 0.07 | 0.618 |
|  | rhTPO 300 vs NAT | 0.12 | 0.07 | 0.104 |
|  | IVIG vs NAT | 0.16 | 0.04 | 0.003 |

rhTPO, recombinant human thrombopoietin; IVIG, intravenous immunoglobulin; NAT, no additional treatment; MD, mean difference; SE, standard error. *Range-standardized APACHE scores (27 for APACHE II and 94 for APACHE III) were used for transitivity assessment.
