# Supplementary figures and images for: A Network Meta-Analysis of Two Doses of Recombinant Human Thrombopoietin for Treating Sepsis-Related Thrombocytopenia
Source: Int J Clin Pract. 2022 Dec 30;2022:2124019. doi: 10.1155/2022/2124019 (PMC9822753; doi:10.1155/2022/2124019)

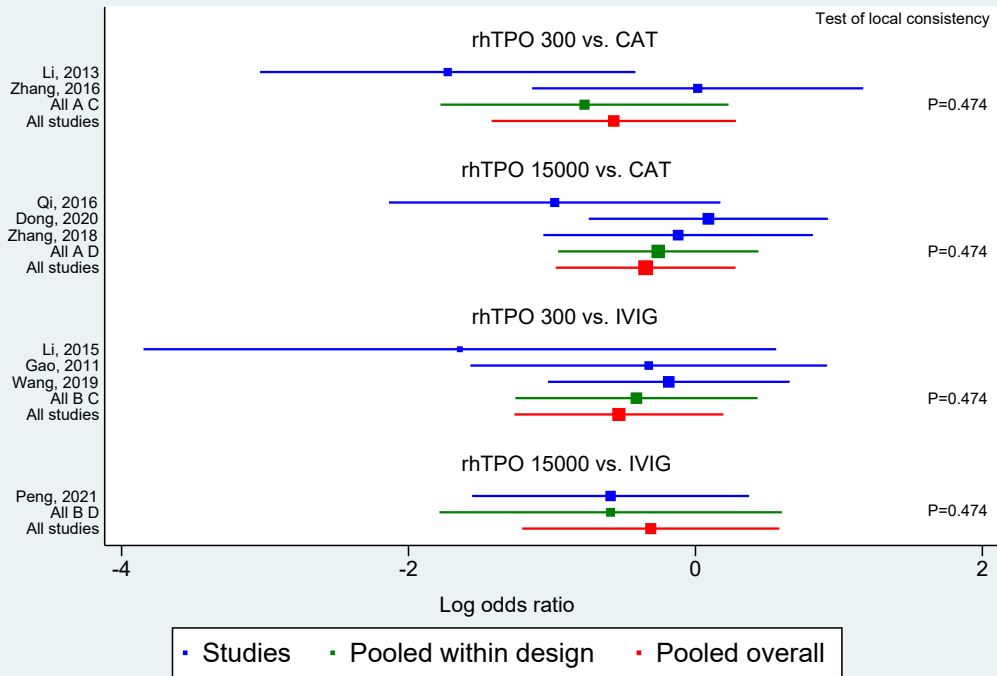

Test of global consistency:  $\chi^2(1)=0.51$ ,  $P=0.474$

Supplement: Supplementary Materials — Search strategy of pubmed, search strategy of Embase, and search strategy of Cochrane library are given. Table S1. Results of individual studies included in this network meta-analysis. Table S2. Transitivity between different comparisons based on major characteristics. Figure S1. Risk of bias summary (a) and graph (b). Red (−), yellow (?), and green (+) color indicates high, unclear, and low risk of bias, respectively. Figure S2. Consistency model test of 28-day mortality. NAT, no additional treatment; IVIG, intravenous immunoglobulin; rhTPO 300, 300 U/kg/d recombinant human thrombopoietin; rhTPO 15000, 15000 U/d recombinant human thrombopoietin. Figure S3. Consistency model test of the length of ICU stay. NAT, no additional treatment; IVIG, intravenous immunoglobulin; rhTPO 300, 300 U/kg/d recombinant human thrombopoietin; rhTPO 15000, 15000 U/d recombinant human thrombopoietin. Figure S4. Evidence network of secondary outcomes including the level of platelet on the 7th day (a), transfusion of RBC (b), transfusion of plasma (c), and transfusion of platelet (d). NAT, no additional treatment; IVIG, intravenous immunoglobulin; rhTPO 300, 300 U/kg/d recombinant human thrombopoietin; rhTPO 15000, 15000 U/d recombinant human thrombopoietin. Figure S5. The consistency model test of the secondary outcomes including the level of platelet on the 7th day (a), transfusion of RBC (b), transfusion of plasma (c), and transfusion of platelet (d). NAT, no additional treatment; IVIG, intravenous immunoglobulin; rhTPO 300, 300 U/kg/d recombinant human thrombopoietin; rhTPO 15000, 15000 U/d recombinant human thrombopoietin. Figure S6. Results of consistency test based on closed-loop. NAT, no additional treatment; IVIG, intravenous immunoglobulin; rhTPO 300, 300 U/kg/d recombinant human thrombopoietin; rhTPO 15000, 15000 U/d recombinant human thrombopoietin; IF, inconsistency factor; CI, confidence interval. Figure S7. Comparison-adjusted funnel plot of 28-day mortality (a) and the len [file 2124019.f1.zip › Figure S2.pdf]

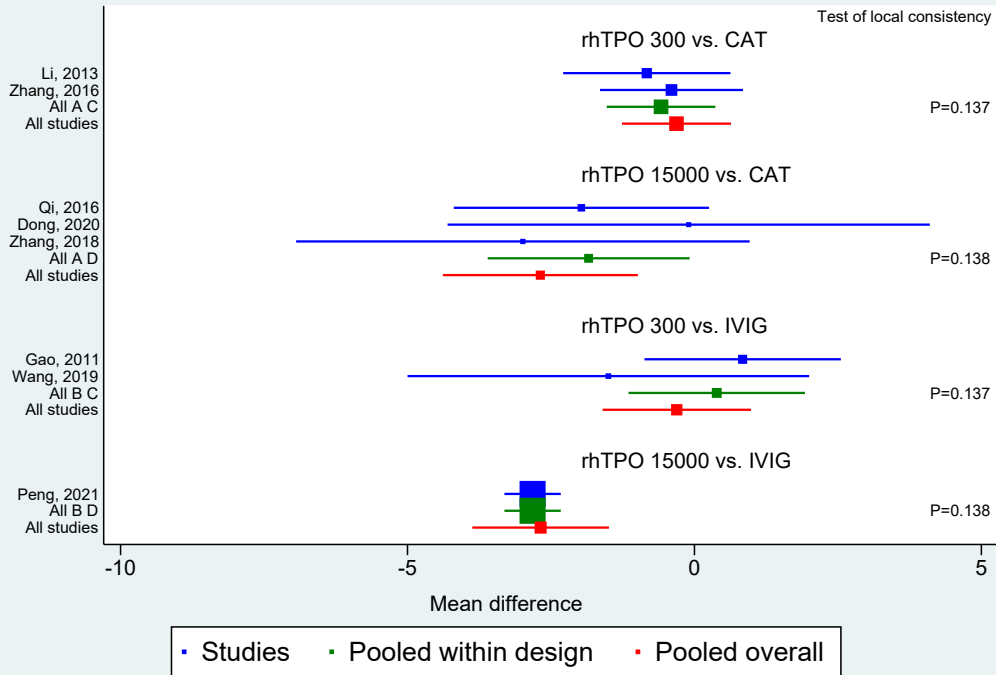

Test of consistency:  $\chi^2(1)=2.21$ ,  $P=0.137$

Supplement: Supplementary Materials — Search strategy of pubmed, search strategy of Embase, and search strategy of Cochrane library are given. Table S1. Results of individual studies included in this network meta-analysis. Table S2. Transitivity between different comparisons based on major characteristics. Figure S1. Risk of bias summary (a) and graph (b). Red (−), yellow (?), and green (+) color indicates high, unclear, and low risk of bias, respectively. Figure S2. Consistency model test of 28-day mortality. NAT, no additional treatment; IVIG, intravenous immunoglobulin; rhTPO 300, 300 U/kg/d recombinant human thrombopoietin; rhTPO 15000, 15000 U/d recombinant human thrombopoietin. Figure S3. Consistency model test of the length of ICU stay. NAT, no additional treatment; IVIG, intravenous immunoglobulin; rhTPO 300, 300 U/kg/d recombinant human thrombopoietin; rhTPO 15000, 15000 U/d recombinant human thrombopoietin. Figure S4. Evidence network of secondary outcomes including the level of platelet on the 7th day (a), transfusion of RBC (b), transfusion of plasma (c), and transfusion of platelet (d). NAT, no additional treatment; IVIG, intravenous immunoglobulin; rhTPO 300, 300 U/kg/d recombinant human thrombopoietin; rhTPO 15000, 15000 U/d recombinant human thrombopoietin. Figure S5. The consistency model test of the secondary outcomes including the level of platelet on the 7th day (a), transfusion of RBC (b), transfusion of plasma (c), and transfusion of platelet (d). NAT, no additional treatment; IVIG, intravenous immunoglobulin; rhTPO 300, 300 U/kg/d recombinant human thrombopoietin; rhTPO 15000, 15000 U/d recombinant human thrombopoietin. Figure S6. Results of consistency test based on closed-loop. NAT, no additional treatment; IVIG, intravenous immunoglobulin; rhTPO 300, 300 U/kg/d recombinant human thrombopoietin; rhTPO 15000, 15000 U/d recombinant human thrombopoietin; IF, inconsistency factor; CI, confidence interval. Figure S7. Comparison-adjusted funnel plot of 28-day mortality (a) and the len [file 2124019.f1.zip › Figure S3.pdf]

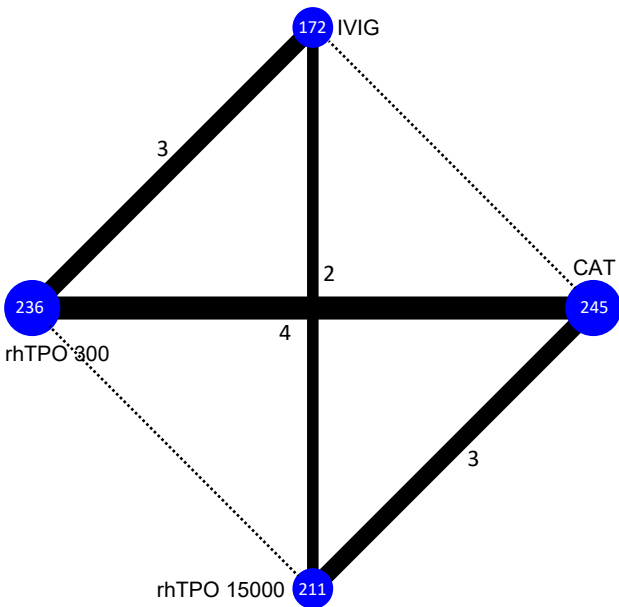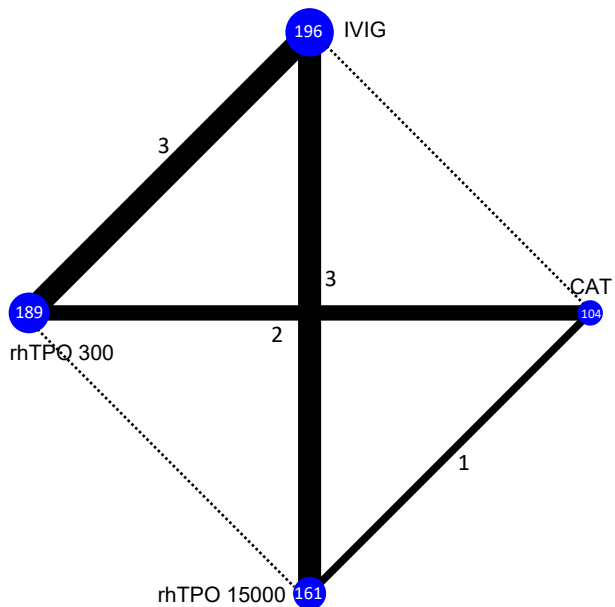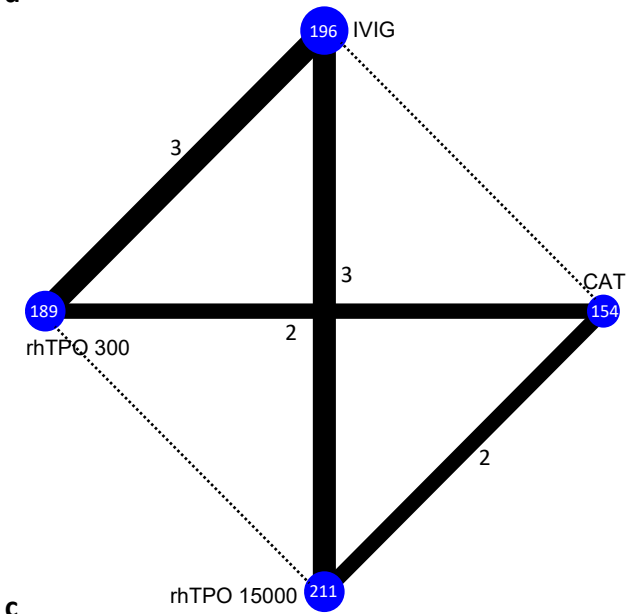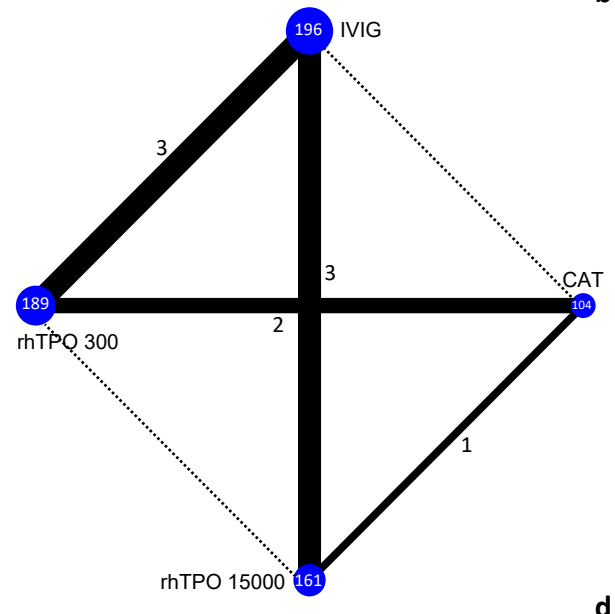

Supplement: Supplementary Materials — Search strategy of pubmed, search strategy of Embase, and search strategy of Cochrane library are given. Table S1. Results of individual studies included in this network meta-analysis. Table S2. Transitivity between different comparisons based on major characteristics. Figure S1. Risk of bias summary (a) and graph (b). Red (−), yellow (?), and green (+) color indicates high, unclear, and low risk of bias, respectively. Figure S2. Consistency model test of 28-day mortality. NAT, no additional treatment; IVIG, intravenous immunoglobulin; rhTPO 300, 300 U/kg/d recombinant human thrombopoietin; rhTPO 15000, 15000 U/d recombinant human thrombopoietin. Figure S3. Consistency model test of the length of ICU stay. NAT, no additional treatment; IVIG, intravenous immunoglobulin; rhTPO 300, 300 U/kg/d recombinant human thrombopoietin; rhTPO 15000, 15000 U/d recombinant human thrombopoietin. Figure S4. Evidence network of secondary outcomes including the level of platelet on the 7th day (a), transfusion of RBC (b), transfusion of plasma (c), and transfusion of platelet (d). NAT, no additional treatment; IVIG, intravenous immunoglobulin; rhTPO 300, 300 U/kg/d recombinant human thrombopoietin; rhTPO 15000, 15000 U/d recombinant human thrombopoietin. Figure S5. The consistency model test of the secondary outcomes including the level of platelet on the 7th day (a), transfusion of RBC (b), transfusion of plasma (c), and transfusion of platelet (d). NAT, no additional treatment; IVIG, intravenous immunoglobulin; rhTPO 300, 300 U/kg/d recombinant human thrombopoietin; rhTPO 15000, 15000 U/d recombinant human thrombopoietin. Figure S6. Results of consistency test based on closed-loop. NAT, no additional treatment; IVIG, intravenous immunoglobulin; rhTPO 300, 300 U/kg/d recombinant human thrombopoietin; rhTPO 15000, 15000 U/d recombinant human thrombopoietin; IF, inconsistency factor; CI, confidence interval. Figure S7. Comparison-adjusted funnel plot of 28-day mortality (a) and the len [file 2124019.f1.zip › Figure S4.pdf]

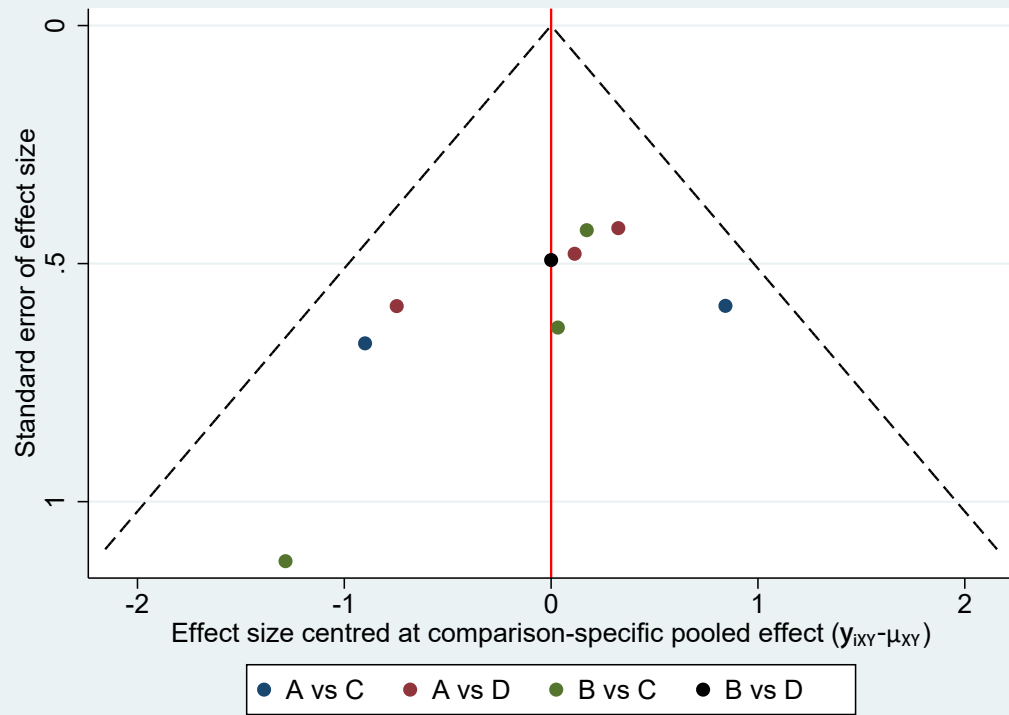

**a**

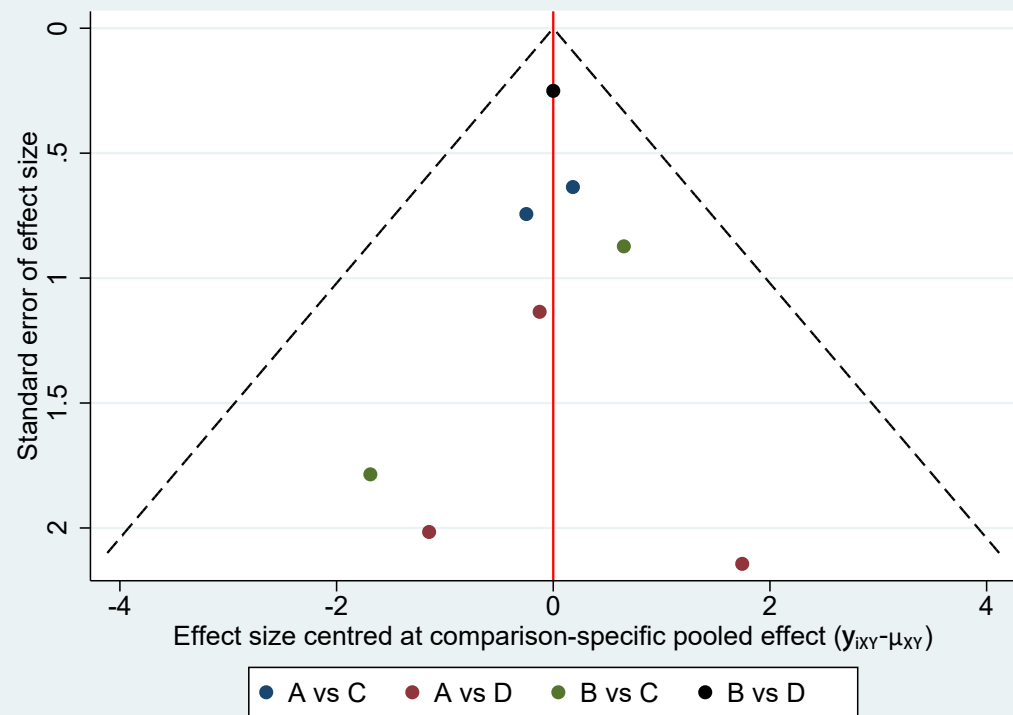

**b**

Supplement: Supplementary Materials — Search strategy of pubmed, search strategy of Embase, and search strategy of Cochrane library are given. Table S1. Results of individual studies included in this network meta-analysis. Table S2. Transitivity between different comparisons based on major characteristics. Figure S1. Risk of bias summary (a) and graph (b). Red (−), yellow (?), and green (+) color indicates high, unclear, and low risk of bias, respectively. Figure S2. Consistency model test of 28-day mortality. NAT, no additional treatment; IVIG, intravenous immunoglobulin; rhTPO 300, 300 U/kg/d recombinant human thrombopoietin; rhTPO 15000, 15000 U/d recombinant human thrombopoietin. Figure S3. Consistency model test of the length of ICU stay. NAT, no additional treatment; IVIG, intravenous immunoglobulin; rhTPO 300, 300 U/kg/d recombinant human thrombopoietin; rhTPO 15000, 15000 U/d recombinant human thrombopoietin. Figure S4. Evidence network of secondary outcomes including the level of platelet on the 7th day (a), transfusion of RBC (b), transfusion of plasma (c), and transfusion of platelet (d). NAT, no additional treatment; IVIG, intravenous immunoglobulin; rhTPO 300, 300 U/kg/d recombinant human thrombopoietin; rhTPO 15000, 15000 U/d recombinant human thrombopoietin. Figure S5. The consistency model test of the secondary outcomes including the level of platelet on the 7th day (a), transfusion of RBC (b), transfusion of plasma (c), and transfusion of platelet (d). NAT, no additional treatment; IVIG, intravenous immunoglobulin; rhTPO 300, 300 U/kg/d recombinant human thrombopoietin; rhTPO 15000, 15000 U/d recombinant human thrombopoietin. Figure S6. Results of consistency test based on closed-loop. NAT, no additional treatment; IVIG, intravenous immunoglobulin; rhTPO 300, 300 U/kg/d recombinant human thrombopoietin; rhTPO 15000, 15000 U/d recombinant human thrombopoietin; IF, inconsistency factor; CI, confidence interval. Figure S7. Comparison-adjusted funnel plot of 28-day mortality (a) and the len [file 2124019.f1.zip › Figure S7.pdf]
